# Impacts of low coverage depths and post-mortem DNA damage on variant calling: a simulation study

**DOI:** 10.1186/s12864-015-1219-8

**Published:** 2015-01-23

**Authors:** Matthew Parks, David Lambert

**Affiliations:** Environmental Futures Research Institute, Griffith University, Nathan, 4111 Queensland Australia

**Keywords:** Ancient DNA, Variant calling, Next-generation sequencing, Coverage depth

## Abstract

**Background:**

Massively parallel sequencing platforms, featuring high throughput and relatively short read lengths, are well suited to ancient DNA (aDNA) studies. Variant identification from short-read alignment could be hindered, however, by low DNA concentrations common to historic samples, which constrain sequencing depths, and post-mortem DNA damage patterns.

**Results:**

We simulated pairs of sequences to act as reference and sample genomes at varied GC contents and divergence levels. Short-read sequence pools were generated from sample sequences, and subjected to varying levels of “post-mortem” damage by adjusting levels of fragmentation and fragmentation biases, transition rates at sequence ends, and sequencing depths. Mapping of sample read pools to reference sequences revealed several trends, including decreased alignment success with increased read length and decreased variant recovery with increased divergence. Variants were generally called with high accuracy, however identification of SNPs (single-nucleotide polymorphisms) was less accurate for high damage/low divergence samples. Modest increases in sequencing depth resulted in rapid gains in total variant recovery, and limited improvements to recovery of heterozygous variants.

**Conclusions:**

This *in silico* study suggests aDNA-associated damage patterns minimally impact variant call accuracy and recovery from short-read alignment, while modest increases in sequencing depth can greatly improve variant recovery.

**Electronic supplementary material:**

The online version of this article (doi:10.1186/s12864-015-1219-8) contains supplementary material, which is available to authorized users.

## Background

The field of ancient DNA (aDNA) has reached 30 years of age, in which time it has progressed from the amplification of fragments of single loci to the complete sequencing of individual complex genomes [1,2]. In its first two decades, aDNA research was primarily focused on PCR-based amplification and subsequent Sanger sequencing of selected loci and organellar genomes, with results applied to analyses of population differentiation and phylogeography, phylogenetics, and even metagenomics (reviewed in [3,4]). In the last decade the field of aDNA has moved from the genetic to the genomic level with the advent of massively parallel sequencing platforms. This has been accompanied by a concurrent shift in focus to full genome sequencing and assembly, and genome-scale analyses of population trends [5-12].

As this field enters its fourth decade, it is inevitable that application of genome-level sequencing will become more commonplace. This will facilitate the broader development of ancient population genomics and phylogenomics approaches [1,2] in a wider range of taxa. Nonetheless, in order to fully benefit from genome-scale approaches, it is critical to understand how the unique characteristics of aDNA might impact results from high-throughput sequencing and read-mapping technologies. In general, there are several important characteristics that distinguish ancient from modern DNA samples. First, not only are aDNA molecules highly fragmented [13], but the fragmentation process itself is biased toward breakpoints bordered by 5′ purine residues (and hence 3′ pyrimidine residues) [14] which could result in biased coverage patterns. Further, deamination of cytosine residues at or near fragment ends ultimately leads to C-T and G-A misincorporations at 5′ and 3′ sequence ends, respectively [15], and could confound aDNA read mapping and variant calling. Last, but not least, ancient samples typically feature relatively low proportions of endogenous DNA [13,16] such that sequencing multiple samples to higher coverage depths (for example >20× depth) to enable confident genomic assembly and variant calling, is often either cost-prohibitive or simply not possible due to limited sample or DNA availability. As a consequence of this, most reports applying high throughput sequencing to full genomes of ancient samples have been limited to one or several samples sequenced and at low or ultra-low (i.e, less than 1×) to moderate coverage depths [2]. This combination can severely impact the accuracy of variant calls [17].

While it is relatively simple to document the biases associated with mapping aDNA sequence reads (for example [18]), it is more difficult to measure the impacts of these biases on variant calling when analysing empirical data. Considering this, we designed a study to quantify the impact of aDNA fragmentation and misincorporation biases on variant calling using simulated data. By utilizing simulated datasets, we were able to control for a number of potentially confounding factors that commonly feature in and could have an effect upon aDNA studies. These include level of divergence between sample and reference, average read length, average sequencing depth and damage level (here designated by levels of fragmentation bias and cytosine deamination at sequence ends). In addition, by synthesizing *in silico* reference and sample sequences prior to incorporation of aDNA-associated damage patterns, it is possible to know the exact “pre-damage” nucleotide sequences of samples. This allows direct comparison of called variants to the true variants, in terms of both accuracy and overall completeness.

## Results

To explore the impacts of post-mortem DNA damage on variant calling, we first simulated triplicate pairs of reference and diploid sample sequences at each of three GC contents (35%, 50%, 65%) and two divergence levels (ca. 0.3% and 3%), and at ca. 10 Mbp (million base pair) lengths, as described in the Methods. Short read sequence pools were artificially generated from the sample sequences at average read lengths of 40, 60 and 80 bp, and were further subjected to either zero, low or high levels of synthetic post-mortem damage reflective of previous reports. This damage included both fragmentation bias and elevated 5′ C-T/3′ G-A transitions near sequence ends. These pools were subsequently mapped back to their corresponding reference sequences using commonly applied alignment software and at a range of ultra-low to moderate sequencing coverage depths (0.1×, 0.5×, 1×, 2×, 4×, 8× and 16×). This method allowed us to directly compare known damage patterns and variant positions to those measured from mapped read pools, and ultimately to estimate whether and to what extent a variety of variables, including coverage depth, read length and damage level, might impact upon variant calling.

### Synthetic damage patterns

Implemented patterns of fragmentation bias were clearly seen when mapping reads with both low and high levels of synthetic damage (Additional file 1). In low damage read pools, 5′ guanosine and adenosine residues were elevated by ca. 20% and 10%, respectively, over genomic levels at positions immediately 5′ to mapped low damage reads. Conversely, guanosine and adenosine residues were diminished by ca. 20% and 10% compared to genomic levels at positions immediately 3′ to mapped low damage reads. The same pattern was seen in mapped high-damage reads, but at ca. 60% and 30% differences from reference genome levels. For both low and high damage read pools, complementary pyrimidine residues at genomic positions immediately 5′ and 3′ of mapped reads showed a pattern directly opposite of purine residues. Fragmentation bias was not evident in mapped reads from no damage read pools.

Misincorporation rates of mapped reads in low and high damage read pools followed expected trends based on patterns of synthetically introduced damage, and were similar to reports from empirical data for 5′ C-T and 3′ G-A transition rates (for example [19,20]) (Additional file 1). Nonetheless, we also found significant differences between expected and observed misincorporation rates. For example, misincorporation rates of mapped reads were generally lower than expected based on the frequencies of incorporated damage in low and high damage treatments (Additional file 2), in some sample replicates dropping to ca. 40% of the expected value at sequence read ends. While misincorporation frequencies at both 5′ and 3′ ends of reads were significantly and positively correlated to damage level (Additional file 2), they were also significantly and negatively correlated to read length at 5′ ends and the 3′-most position of mapped reads (Additional file 2). In addition, 5′ C-T misincorporations were slightly but significantly less common than 3′ G-A misincorporations, with 5′ C-T misincorporations averaging ca. 84-98% of the frequency of 3′ G-A misincorporations across divergence and damage levels (Additional file 2).

### Read mappability

**Figure 1:**
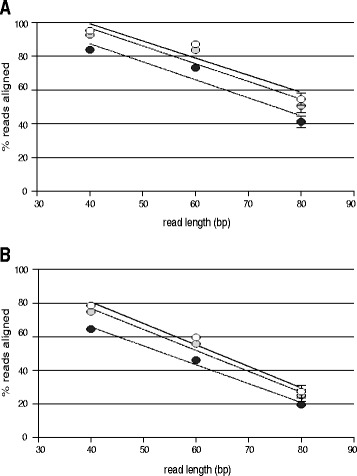
**Average proportions of simulated read pools mapped to references across treatments. A)** Low divergence level and **B)** high divergence level. White, grey and black circles represent no damage, low damage and high damage treatments, respectively. Error bars represent standard deviations of measurements. For A) and B), best-fit linear relationship is shown for no damage (solid line), low damage (dashed line) and high damage (dashed/dotted line) samples; slopes for linear relationships in A) and B) range from -1.03 to -1.29; R^2^ values range from A) 0.89 to 0.92 and B) 0.98 to 0.99 (see Additional file 3 for full details).

GC content, read length and damage all had significant impacts on the proportion of read pools able to be mapped back to their reference at both levels of divergence, while coverage depth was not significantly correlated for either low or high divergence read pool mapping (Table 1). Read length clearly had the most dramatic impact on read mappability, however, and decreases in successful read mapping were strongly correlated with increasing read length for all damage levels and GC contents at both levels of divergence (Figure 1, Table 1). The rate of decrease in read mapping success was very similar for low and high divergence read pools (Table 1, Additional file 3). Nonetheless, the overall lower success rate of read mapping in high divergence read pools resulted in an approximately three-fold decrease in the percent of reads mapped when read lengths were increased from 40 to 80 bp, as opposed to an approximately two-fold decrease observed in low divergence pools. Increasing average read length of a read pool also resulted in a greater decrease in average length of mapped reads across all damage levels and at both low and high divergence (Pr <<0.001 for linear regression), such that average lengths of mapped reads at 40/60/80 bp expected size were 40.05/59.99/79.44 and 40.03/59.92/79.16 bp for low and high divergence read pools, respectively. The impact of damage levels on read mappability was also clear, albeit lesser than that of read length, as the average percent of reads mapped decreased with increased damage at all read lengths, for both divergence levels (Figure 1, Table 1).

**Table 1:** **Impact of independent variables on the percent of reads mapped from read pools**

**Divergence**	**%GC**	**Read length**	**Damage**	**Coverage depth**
Low	-0.1212*	-0.7032***	-6.4180***	-0.0005
High	-0.1068*	-0.8363***	-5.9020***	0.0008

Slope coefficients resulting from multiple variable regression analysis are shown applying best-fit linear models, considering as dependent variables the percent of reads mapped from a pool, while GC content, read length, damage level and coverage depth were treated as independent variables. Values of 1, 2 and 3 were assigned to variables no-, low- and high-damage, respectively. Values not significant at Pr < 0.05 unless otherwise indicated; * signifies 0.01 < Pr < =0.05; *** signifies Pr < =0.001.

### Accuracy of variant calls

**Figure 2:**
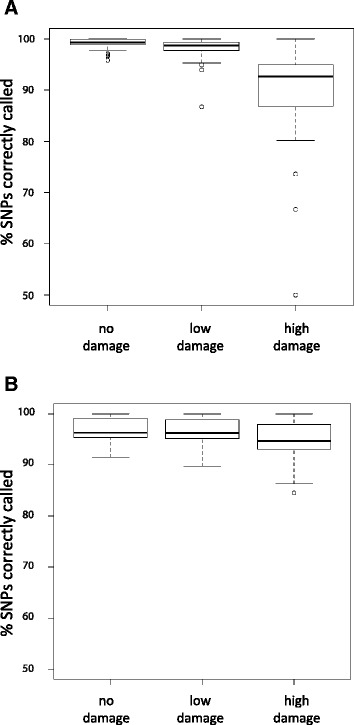
**Effects of damage on accuracy of SNP calls across all triplicate-averaged read pools.** Box and whiskers plots for **A)** low and **B)** high divergence levels.

Both SNPs (single nucleotide polymorphisms) and indels (insertions-deletions) were generally called with high accuracy across coverage depths, GC contents, read lengths, and damage levels. Indels averaged 95.4% (sd = 2.5) and 92.4% (sd = 2.2) correct, and SNPs averaged 91.7% (sd = 11.6) and 97.8% (sd = 2.4) correct, for low and high divergence treatments, respectively. Nonetheless, SNP and indel call accuracy were differentially impacted by damage levels. The accuracy of indel calls was not substantially affected by damage level at either level of divergence (Table 2). In contrast, SNP call accuracy was diminished by higher rates of damage, particularly at low divergence (Table 2, Figure 2). A significant and positive correlation between read length and variant call accuracy was also demonstrated for both SNPs and indels in high divergence treatments, and for SNPs in low divergence treatments, although the impact of this relationship was relatively small (Table 2). GC content had significant correlations to variant call accuracy only for SNP calls (negative relationship) at high divergence (Table 2); the magnitude of this relationship was also relatively small.

**Table 2:** **Impact of independent variables on variant call accuracy and variant call completeness**

**Divergence**		**%GC**	**Read length**	**Damage**	**Coverage depth**
Low	% indels called correctly	-0.0194	0.0153	0.2150	0.4723log(covdepth)***/-0.1687***
	% SNPs called correctly	-0.0757	0.0828*	-5.1790***	-0.2730log(covdepth)/0.0405
	% of total indels called	-0.0459	-0.1057***	-2.7754**	3.9708***
	% of total SNPs called	-0.0314	-0.0521	-1.6778*	5.5177***
High	% indels called correctly	0.0092	0.0396***	0.2563*	1.660log(covdepth)***/-0.3292***
	% SNPs called correctly	-0.1495***	0.0249***	-0.9437***	-0.2730***
	% of total indels called	-0.0282	-0.1159***	-1.8524***	2.8565***
	% of total SNPs called	-0.0867**	-0.0616**	-1.0032*	3.1498***

Slope coefficients resulting from multiple variable regression analysis, considering as dependent variables the percent of indels/SNPs correctly called (variant call accuracy) and the percent of total indels/SNPs called (variant call completeness), while GC content, read length, damage level and coverage depth were treated as independent variables. Values of 1, 2 and 3 were assigned to variables no-, low- and high-damage, respectively. Values not significant at Pr < 0.05 unless otherwise indicated; * signifies 0.01 < Pr < =0.05; ** signifies 0.001 < Pr < =0.01; *** signifies Pr < =0.001. For each row, the independent variable with the strongest significant effect is highlighted in bold. Results solely for best-fit linear models are shown except for “%indels called correctly” at low and high divergence and “% SNPs called correctly” at low divergence. For these cases, a slight improvement in correlation was seen using a logarithmic model, and slope coefficients for both models are shown as: log coefficient / linear coefficient.

### Completeness of variant recovery

**Figure 3:**
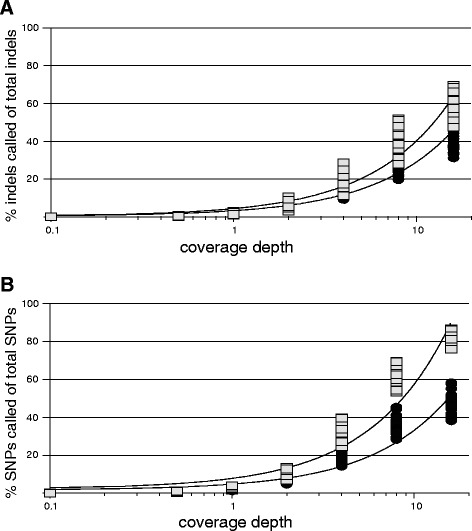
**Effects of coverage depth at low and high divergence levels on variant call completeness. A)** Percent of indels called correctly out of all indels for a reference-sample pair; **B)** Percent of SNPs called correctly out of all SNPs for a reference-sample pair. Squares and circles represent data points for low and high divergence samples, respectively. Best-fit linear relationships shown for low (solid lines) and high (dashed lines) divergence samples for A) and B). Equations and R^2^ values for best-fit linear relationships are A) y = 3.9704x + 0.4293, R^2^ = 0.92759 (low divergence); y = 2.8566x + 0.411, R^2^ = 0.91455 (high divergence); B) y = 5.5174x + 2.3014, R^2^ = 0.92406 (low divergence); y = 3.1491x + 1.6574, R^2^ = 0.90764 (high divergence). Slopes for low versus high divergence samples are not significantly different in A) or B) at P < 0.05; however, percentage of variants found regressed on divergence level is significant at P < 0.05 and P < 0.001 for A) and B), respectively.

In contrast to variant call accuracy, variant call completeness was substantially impacted by coverage depth at both levels of divergence for both SNPs and indels (Table 2, Figure 3). For example, SNP calls in low divergence samples varied from ca. 0.01% complete at 0.1× coverage depth to nearly 81.55% complete at 16× coverage depth. This trend was not quite as pronounced at high divergence, as on average SNP recovery ranged from ca. 0% (0.1× coverage depth) to ca. 47% (16× coverage depth) for high divergence read pools. Indel call completeness was lower on average than SNP call completeness for low divergence read pools, peaking at ca. 59% completeness for 16× coverage depth, but was nearly indistinguishable from SNP call completeness for high divergence read pools. Both SNP and indel call completeness at low and high divergence were also significantly and negatively correlated with read length, with the exception of SNP call completeness at low divergence (negative relationship, but not significant) (Table 2). However, this impact was more than an order of magnitude smaller than that of coverage depth in all cases. Damage level had a significant and negative influence on variant call completeness for both SNPs and indels, although this relationship did not have as strong an impact as coverage depth (Table 2).

### Recovery of homozygous versus heterozygous variants

**Figure 4:**
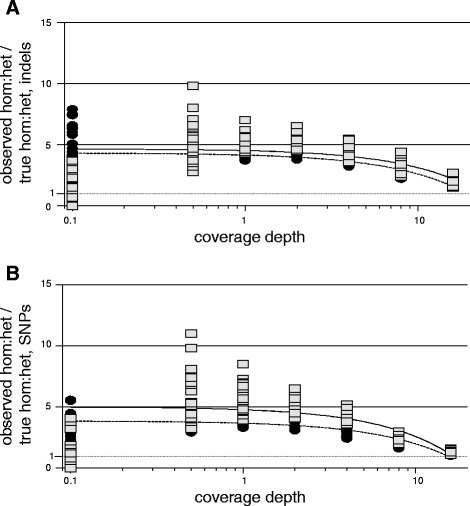
**Effects of coverage depth on ratio of homozygous to heterozygous variant calls.** Ratios of observed homozygous:heterozygous to true homozygous:heterozygous variant call ratios. Squares and circles represent data points for low and high divergence samples, respectively, for **A)** indel variant calls and **B)** SNP variant calls. Equations and R^2^ values for best-fit linear relationship are **A)** y = -0.1288 + 4.3862, R^2^ = 0.15353 for low divergence samples (solid line); y = -0.1712x + 4.3267, R^2^ = 0.65332 for high divergence samples (dashed line); **B)** y = -0.1899x + 4.4411, R^2^ = 0.21871 for low divergence samples (solid line); y = -0.1835x + 3.8176, R^2^ = 0.64356 for high divergence samples (dashed line). Dashed grey line added at unity for observed hom:het / true hom:het for visual clarity.

We observed for both SNP and indel recovery at both levels of divergence that ratios of homozygous to heterozygous variant calls approached their expected values with increasing coverage depth (Figure 4, Additional file 4), but closely approached expected values only for SNPs at the highest coverage depth tested (16×). Neither GC content, read length or damage level significantly affected ratios of homozygous to heterozygous variant calls, with the exception of a slight negative correlation between read length and SNP homozygous to heterozygous ratios at high divergence. The impact of read length in this case was more than an order of magnitude less than that of coverage depth.

## Discussion

As aDNA research broadens its utilization of genome-level sequencing, it is essential to understand how the unique characteristics of aDNA molecules might impact variant calling from short-read sequence data sets generated by massively parallel sequencing platforms. In the current study, we quantified the impact of several variables, including the level of divergence between reference and sample sequences, levels of damage in “ancient” sample sequences, and coverage depth on variant call accuracy and completeness. Our use of simulated datasets enabled precise and relatively unbiased measurements of the effects of aDNA-associated damage on read mapping and variant calling through comparison of known variants to variants called from mapping of short reads with calculated damage patterns. The variable settings used in this study, including the divergence level between reference and sample sequences, sequencing coverage depth and damage patterns, were chosen based on previously reported aDNA studies. For example, in some cases highly similar, same-species reference genomes are available for read mapping for aDNA samples [1,8,21]. Our low divergence reference-sample pairs, at approximately 0.3% divergence, reflect this scenario. Alternatively, in many cases it is necessary to apply more divergent reference genomes from extant genus- or family-level congeners when sequencing extinct taxa [12,22]. Our high divergence reference-sample pairs, at approximately 3% divergence, were used to estimate these effects. In addition, results from these divergence levels may be used as proxy for predicting variant call success across regions of varying evolutionary rates within the same genome. Importantly, the divergence levels (and misincorporation frequencies) employed in this study also capture the challenges associated with mapping reads harbouring multiple variant positions, as sequence reads from high divergence read pools averaged more than one variant position per read length. GC content, while not expected to have an effect on variant calling *a priori*, was also varied in order to reflect the range of conditions that could be encountered across a nuclear genome, or when sequencing organellar, prokaryotic or viral genomes.

Levels of fragmentation, fragmentation biases, and misincorporation rates used in the present study were also based on previously reported aDNA damage levels. Fragmentation is a universal process associated with post-mortem DNA degradation [23,24], with the result that average read lengths reported in aDNA studies are typically under 100 bp, and can range to under 50 bp [24]. In turn, nucleotide frequencies at positions immediately adjacent to fragment ends can shift by approximately 10% to over 70% of expected values due to fragmentation bias (for example [20,25]). Although it may seem intuitive that levels of fragmentation and fragmentation bias would increase with sample age and level of damage, as misincorporation rates do, these relationships do not hold consistently, and may in some cases be reversed [26]. Nonetheless, our goal was to both encapsulate typical variation in aDNA damage levels, as well as model “worse-case” scenarios for comparative purposes. Similarly, the misincorporation frequencies used in this study were within the range of reported values for a range of aDNA samples. Misincorporation frequencies for late Pleistocene Neandertal, cave bear and mammoth sequences have been reported in the range of ca. 0.2-0.25 at sequence read ends [27], while over 0.3 for a Holocene human sample [10], and as low as ca. 0.05-0.1 for a quagga museum specimen and a late Pleistocene *Hippidion* fossil [28].

Importantly, although our damage levels fell within those previously reported in a range of studies, our data suggest that fragmentation and deamination patterns of aDNA molecules can result in misrepresentation of the characteristics of aDNA read pools as reported solely from mapped reads. For example, read length, deamination rates at fragment ends, and increased divergence to reference all substantially impacted the efficiency of read mapping. As a result, the proportion of sequence reads mapped successfully from a given read pool ranged from over 90% to under 20% (Figure 1), which in turn would result in up to ca. five-fold under-estimation of the endogenous content of a read pool. Similarly, misincorporation rates recorded from mapped read pools tended to be lower than values set in read pools prior to mapping, and across treatments were significantly and negatively correlated to read length (Additional file 2). Taken together, these results further suggest that the loss of information due to unsuccessful read mapping of aDNA fragments can in some cases be substantial. In our study, this loss was exacerbated by relatively longer read lengths, increased divergence between reference and sample sequences and by increased misincorporation damage near the ends of sequence reads. It is possible that the utilization of alternative mapping and variant calling pipelines could also impact these results. In particular, other short read alignment algorithms designed for higher sensitivity, such as SOAP [29], BWA-MEM (unpublished, http://bio-bwa.sourceforge.net/bwa.shtml) or BWA-PSSM [30], might improve read mapping or variant call performance, particularly at higher divergence levels [31]. Alternatively, adjusting BWA mapping parameters alone could improve read mapping success rates in some cases. However, exploration of BWA parameters on Illumina aDNA sequence reads has been shown to result in very small gains (ca. 1%) in the number of reads mapped to a reference [32], and alignment algorithms beyond BWA have not yet been widely applied to aDNA studies. Finally, the magnitude of decrease in mapped reads with longer read lengths is likely dependent to some degree on the read-simulation algorithm used in this study [33], and so might also vary with different reference-sample combinations and the sequencing platform employed in aDNA studies.

Our results also suggest that damage patterns associated with aDNA molecules may often have relatively little impact on variant call accuracy at low to moderate average coverage depths. Simulated indels, and to a slightly lesser extent, SNPs, were generally called accurately across read lengths and damage levels, and the magnitude of the impact of damage on variant call accuracy was small for indels. The absolute values for variant call accuracy reported here are also likely affected by the use of simulated data (both sequences and sequencing data) and the applied variant calling pipeline, and may be higher in biological datasets or with alternative bioinformatic strategies (for example, see [34]). However, it is also clear that SNP call accuracy is impacted at high damage levels. This trend is reinforced by the significant and positive correlation between damage level and misincorporation frequency in mapped reads (Additional file 2). It is somewhat surprising that the impact of damage on SNP call accuracy was more pronounced at a lower level of divergence (Figure 2). It is likely in these cases that uneven coverage (due to fragmentation bias) coupled with relatively higher levels of misincorporations, resulted in sufficient numbers of reads carrying identical ‘post-mortem’ damage to give erroneous SNP calls. In support of this, we found significant negative correlations (Pr <<0.001 from linear regression) between damage levels and the proportion of mapped reads in a pool with unique 5′ mapping positions at both low and high divergence levels, suggesting that increased fragmentation bias (and possibly misincorporation bias) does lead to uneven distribution of mapped reads across a reference genome. This trend may be lessened at higher divergence, as the introduction of high levels of ‘post-mortem’ damage into reads already containing multiple variants further decreases the probability of mapping reads with misincorporations. At the same time, the significant and positive correlations between read length and variant call accuracy for both SNPs and indels (Table 2) further underscore the potential for loss of information through unsuccessful mapping of longer and/or damaged reads noted above. In this regard, it is also worth noting that a high proportion of the lowest triplicate-averaged variant call accuracy scores came from read pools with the shortest average read lengths. For example, among SNP and indel call accuracy scores less than 90 percent, approximately two thirds were from read pools with 40 bp average read lengths (18/26 and 1/2 for SNPs and indels, respectively, in low divergence pools; 5/8 and 13/19 for SNPs and indels in high divergence pools).

We also found that variant call accuracy and coverage depth were significantly correlated, particularly at high divergence (Table 2). Unexpectedly, this relationship was negative, suggesting that under the variant calling algorithm applied here, variant call accuracy can suffer slightly as coverage depth increases across low coverage levels. This impact was relatively minor, however, and was approximately an order of magnitude smaller than concurrent percentage gains in overall variant recovery (discussed below). In addition, we found the negative impact of increasing coverage depth on variant call accuracy was diminished when considering coverage depths greater than 1×, suggesting that optimal gains in variant calls may be encountered by increasing sample coverage from low to moderate levels as opposed to, for example, increasing coverage depths from ultra-low to low levels.

Similar to variant call accuracy, overall variant recovery was not strongly impacted by damage levels, although we did find significant and negative relationships between damage and variant call completeness for both SNPs and indels (Table 2). In contrast, coverage depth clearly had a substantial impact on the completeness of variant recovery, with recovery increasing by ca. 2.8-5.5× for SNPs and indels for each 1× increase in coverage depth (Figure 3). Slight negative correlations between variant call completeness and read length (Table 2) again likely reflect the difficulty in mapping longer reads, which results in lower coverage depth and decreased variant recovery. In addition, divergence clearly impacted variant call completeness for both SNPs and indels (Figure 3), in particular at high coverage levels, suggesting that the availability of a closely related reference may be of substantial benefit in this regard.

As a general trend, the lower overall recovery rate for indels compared to SNPs evident in our low divergence results is not unexpected. The combination of short read lengths and the requirement for efficiency in alignment algorithms leads to a general bias in calling small polymorphisms, such that confidently called SNPs are overrepresented relative to small insertions and deletions [35,36]. At the same time, small indels have been implicated in phenotype development in a range of organisms [37-42], and may play a stronger role than SNPs in early divergence of closely related taxa [43,44]. Considering this, and the fact that phenotype interrogation will likely become more prevalent with increased genomic sequencing of ancient samples [8,10,45], researchers may consider increasing sequencing efforts beyond that required for efficient SNP recovery in order to more fully capture small indels present in ancient genomes. It is worth noting, however, that a logarithmic correlation between variant call completeness and sequencing coverage depth was nearly as well supported as the best-fitting linear relationships. This suggests that there may be a point of diminishing returns for variant recovery in many cases.

Finally, relative recovery rates of homozygous and heterozygous variants were significantly impacted by coverage depth for both SNPs and indels, with observed rates more closely reflecting expected rates as coverage depths increased (Figure 4, Additional file 4). Nonetheless, ratios of homozygous to heterozygous variants closely approached expected values only for SNPs at 16× coverage depth. The trends in homozygous versus heterozygous variant call success in our analyses suggest that the application of population demographic algorithms based on patterns of heterozygosity (for example [46,47]) may be challenging for aDNA samples at low average coverage depths.

## Conclusions

Overall, the trends in our data are promising for aDNA studies, as increasing sequencing capacity of high-throughput platforms and decreased per bp sequencing costs [48] will continue to enable both broader and deeper sequencing of ancient samples. In addition, targeted enrichment strategies, either focused on sub-genomic targets [21,49] or entire genomes [50], may further increase sequencing efficiency and enable greater per-sample coverage depths. Nonetheless, it is clear that under some circumstances variant calling is significantly impacted by post-mortem damage patterns and low DNA availability typical of aDNA samples, particularly so for accuracy in SNP calls at high levels of damage, and for overall indel and SNP recovery at low coverage depths. Further, evolutionary analyses requiring accurate identification of homozygous versus heterozygous variants are likely to suffer when incorporating low coverage aDNA variant calls. Considering that aDNA-associated damage patterns are essentially unavoidable yet quantifiable features of ancient samples, it is recommended that researchers carefully consider read mapping and variant calling strategies individually for different projects or even for different samples within a project. Further, it is likely that relatively recent efforts in recalibration software based on aDNA damage patterns [51,52] may also aid in increasing variant call accuracy, and ideally will continue to be developed and find broad application in the future.

## Methods

### Generation of divergent reference-sample sequence pairs

Pairs of divergent sequences of moderate size (ca. 10,001,000 base pairs (bp) aligned length) were created using INDELible v1.03 [53] to represent reference sequences and diploid sample sequences. We applied moderate settings for sequence evolution, using the HKY substitution model [54] with transition probabilities set at twice that of transversion probabilities, the gamma distribution parameter set at 1.0 with four discrete rate categories, and the proportion of invariant sites set to 0.1. Indels were simulated under the power law setting, with a = 1.7 and a maximum length (M) of 5 bp. Since INDELible does not directly simulate diploid sequence evolution, we approximated diploid sample sequences by adding a bifurcation event halfway along each sample’s divergence from the reference lineage in the user-specified tree (i.e., (reference:1x,(chromosome1:0.5x,chromosome2:0.5x):0.5x)). The resulting sequence pair was treated as homologous chromosomes. This synthesis was done in triplicate for each of three GC content levels (35%, 50%, and 65% GC content) at two levels of divergence, resulting in six sets of triplicate sequence pairs, or 18 total sequence pairs. Divergence levels were set such that positions were affected by SNPs and indels at approximately equal frequencies, with on average ca. 2.6 variant positions per 1000 bp for “low” divergence and 2.9 variant events per 100 bp for “high” divergence sequence pairs. Under the read length distributions used in this study (see below), this resulted in, on average, approximately 0.11-0.21 variant positions per read length for low divergence read pools, and 1.2-2.3 variant positions per read length for high divergence read pools. Resultant indel lengths averaged 1.84 bp (standard deviation (sd) = 0.02).

### Generation of read pools and damage patterns from sample sequences

From each “chromosome” of each diploid sample sequence, a pool of 100 bp single-end Illumina sequence reads was synthesized to 300× coverage using ART v1.5.0 (art_Illumina Q version) [55] under default settings. Illumina sequence reads were generated, as the Illumina platforms are currently the most commonly used high-throughput platforms [56], and their combination of throughput and read length are well-suited to aDNA sequencing [57]. Read pools for homologous chromosome pairs were combined and randomly ordered, and reads were then drawn repeatedly from these pools and subjected to different treatments representing all permutations of damage levels and coverage depths considered in this study, as described below and illustrated in Additional file 5.

First, the reads from a pool were trimmed to represent read size distributions reflective of highly fragmented ancient DNA. For this purpose, reads were trimmed to fit exponential size distributions spanning 30-50 bp, 50-70 bp or 70-90 bp in length, with average lengths of 40 bp, 60 bp or 80 bp, respectively. For each size distribution, 15% of trimmed reads were equal in size to the average read length value, and represented the largest group of reads; the subsequent proportions of larger or smaller reads in a pool decreased by 25% for each single bp change in read length.

Next, the reads from each trimmed read pool were subjected to “no”, “low”, and “high” damage treatments to incorporate fragmentation biases. Read pools in low and high damage treatments were designed to reflect fragmentation bias toward purine residues immediately prior (i.e., 5′) to read starts and pyrimidine residues immediately after (i.e., 3′ to) read ends. In addition, fragmentation adjacent to guanosine residues was favoured over fragmentation adjacent to adenosine residues at 5′ read ends (and similarly for cytosine versus thymine residues adjacent to 3′ read ends) by a factor of approximately two, based on previous reports [25,27,58]. Detailed frequencies for all combinations of bordering nucleotides are shown in Additional file 6. For no damage read pools, trimmed reads were chosen with a probability of 0.5, without bias based on adjacent sample sequence positions.

Transition misincorporations were then added to reads in the low and high damage read pools, such that 5′ and 3′ ends of reads had elevated C-T and G-A transitions, respectively. The probability of misincorporation was also based on an exponential distribution to approximate the increasing probability of deamination events closer to sequence ends, with the highest probability of misincorporation at the 5′- and 3′-most positions of reads, and each position further away from sequence ends having 75% of the probability of a transition damage as the previous position. Low and high damage read pools were differentiated by the probability of misincorporation at each position and by the total number of positions in reads potentially affected by misincorporations. Reads starting with a cytosine in low damage pools had 0.12 probability of C-T transition, while similar reads in high damage pools had 0.4 probability of transition. 5′ C-T and 3′ G-A transitions were allowed in sequence reads until the probability of transition fell below 0.01, at which point transitions were no longer purposefully incorporated into sequence reads. This resulted in transitions present in the first and last nine positions and first and last thirteen positions of reads for low and high damage pools, respectively.

Last, reads from each treatment were randomly drawn to reach seven different levels of coverage depth in mapping to their original reference sequence, representing a range of ultra-low to moderate coverage depths for Illumina sequencing platforms: 0.1×, 0.5×, 1×, 2×, 4×, 8× and 16×. In total, permutations of the original 18 reference-sample sequence pairs (3× replicates at 3× GC contents and 2× divergence levels, with 3× read length distributions, 3× damage levels and 7× coverage depths) presented 378 sets of triplicate read pools, or 1134 read pools in total. Sequence read pools and the scripts/commands used to generate damage patterns are available upon request from the authors.

### Read mapping and variant calling

The original reference sequences associated with sample read pools were indexed using BWA v0.6.2 [59] and Picard Tools v1.96 (http://broadinstitute.github.io/picard/) with default settings. Read pools were mapped to their corresponding reference using BWA v0.6.2, SAMtools v0.1.18 [60] and GATK v2.4-9-g532efad [61]. We chose this suite of software as BWA and SAMtools are commonly used to map reads from high-throughput aDNA sequencing [11,25,32,62-65] (but see [5,8]). Further, these softwares are amenable to forming efficient and automatable pipelines for genome-scale alignments and variant-calling [52]. For read mapping, the BWA ‘aln’ and ‘samse’ modules were used under default parameter settings except that read seeding was disabled for BWA aln (-l 1024). For alignment manipulation in SAMtools, the following modules were used: view (parameter setting -bS), sort (default parameters), index (default parameters), flagstat (default parameters) and mpileup (parameter setting -Bg). The ‘PrintReads’, ‘RealignerTargetCreator’ and ‘IndelRealigner’ modules of GATK were also used under default settings for indel mapping adjustments. Variant calls were filtered using the bcftools view module of SAMtools (parameter setting -LNcgv), and the varFilter module of vcfutils.pl from SAMtools, specifying minimum and maximum depths of 1 and 30, respectively. Variant calls were initially filtered for quality using four different quality cutoff levels: 1) variant call quality ≥ 20, 2) both variant call quality and genotype quality ≥ 20, 3) variant call quality ≥ 30, and 4) both variant call quality and genotype quality ≥ 30. For SNP variant calls, essentially no difference was found in variant call success between quality filters 1-3, while quality filter 4 performed slightly worse (as quantified by variant call accuracy and completeness) across damage treatments and for both levels of divergence. For indel variant calls, quality filter 1 was the most consistent for variant call success across damage treatments and for both levels of divergence, and either the best-performing quality filter or only slightly less successful in accuracy than the other quality filters. For this reason, only results from quality filter 1 are presented. Potential duplicate reads were not flagged or removed from alignments, as the random nature of read selection and trimming to size distributions for all read pools, and misincorporation events for damage read pools, resulted in highly diverse read pools (average percentage of unique sequences for reads in a read pool = 99.60%, sd = 0.50%). Further, since duplicate marking softwares, such as Picard Tools, designate duplicates simply based on the 5′-most position of mapped reads, it was estimated that duplicate removal would eliminate a greater proportion of reads with unique sequences than true duplicate (i.e., identical) reads. An example set of mapping and variant call commands is given in Additional file 7. Fragmentation bias and misincorporation rates were evaluated from mapping of sample read pools using mapDamage v2.0, under default parameters but with maximum read lengths set to 90 bp (-l 90) [18].

Original reference-sample sequence pairs were aligned using MAVID v.2.0.4 [66] under default parameters and with the reiterative option (-r). Variant calls were directly identified from alignments using custom scripts and were then compared to variant calls from read mapping. Only variant calls mapping between positions 500 to 10000500 of the reference were recorded to negate any potential difficulties of read mapping to ends of reference sequences, resulting in sample sequence lengths of ca. 10 Mbp. Due to the potential for slight variances in indel placement between MAVID and BWA/GATK alignments, indel events identified in read pool mapping were considered called correctly if they occurred within three base pairs of an indel event identified in the reference-sample alignment. SNPs were more stringently compared between alignments, and were considered called correctly if both their position and base call matched. SNP calls were also filtered to include only those occurring >5 bp distant from indel positions. Heterozygous variant calls were treated as separate calls to the same position, such that a heterozygous variant position could contribute either one correctly called and one incorrectly called variant, or two incorrectly called variants, to the tallying process.

### Statistical analyses of variant call counts

We considered the impacts of four variables (GC content, read length, damage level and coverage depth) on read mapping and variant identification for mapped read pools. For each sample read pool, variant calls from read pool mapping were summarized both in terms of their accuracy:

# SNPs or indels correctly called/total # SNPs or indels called for a sample and completeness:

# SNPs or indels correctly called/total # SNPs or indels present in reference-sample pair

For these calculations, again only variant calls mapping to positions 500-10000500 of reference sequences were included in analyses.

To estimate the effectiveness of homozygous versus heterozygous variant recovery, the ratio of correctly called homozygous variants to correctly called heterozygous variants was calculated for SNPs and indels for each read pool. This ratio was compared to the expected homozygous:heterozygous ratio as measured from original reference-sample alignments, such that:

observed homozygous:heterozygous ratio in correctly called variants/expected homozygous:heterozygous variant call ratio.

ideally would approach unity for both SNPs and indels with effective variant calling.

From these variant call measurements for the 1134 sample read pools, triplicate results were averaged, resulting in 378 final data points. The magnitude and significance of impacts on read mappability and variant calling were determined using multiple variable regression and ANCOVA in R v3.0.2 (http://www.r-project.org/). In order to present a clearer view of trends at different levels of divergence, analyses were performed on high and low divergence read pools separately rather than considering divergence level directly as an independent variable. In addition, damage levels were assigned values of 1, 2 and 3 for no, low and high damage read pools, respectively, for statistical analyses.

## Additional files

Additional file 1:
**Examples of typical fragmentation bias and misincorporation patterns.** Example fragmentation and misincorporation patterns for A) low damage sample read pool, B) high damage read pool and C) undamaged read pool. Charts shown are for sample read pool with 50% GC content and 60 bp average read length. Analyses performed using mapDamage v2.0. Additional file 2:
**Summaries of misincorporation frequencies of mapped reads.** A) Ratios of observed versus expected misincorporation rates at 5′ and 3′ sequence ends. Expected values were based on frequency of transition damages incorporated during read synthesis (see Methods). ** signifies confidence interval of value does not include 1 at 0.001 < Pr < 0.01; *** signifies confidence interval of value does not include 1 at Pr < 0.001; remaining values not significant at Pr < 0.05. B) Slope coefficients from multiple variable regression are shown, treating as dependent variables the average misincorporation frequencies for mapped reads at all 5′ and 3′ sequence read positions with elevated misincorporation frequencies (all elevated) or solely the 5′- or 3′-most positions of reads; GC content, read length, damage level and coverage depth are independent variables. Values of 1, 2 and 3 were assigned to no-, low- and high-damage read pools, respectively. Values not significant at Pr < 0.05 unless otherwise indicated; * signifies 0.01 < Pr < =0.05; *** signifies Pr < =0.001. Results in B) are shown solely for best-fit linear models except for “damage” for 5′ and 3′ “all elevated positions” at both low and high divergence. For these cases, a slight improvement in correlation was seen using an exponential model, and slope coefficients for both models are shown as: exponential coefficient/linear coefficient. Additional file 3:
**Regression of read length on percent of reads mapped at different damage and divergence levels.** Slopes of linear regressions and R^2^ values are shown, considering as the dependent variable the percent of reads mapped from a pool, and read length treated as independent variable. Additional file 4:
**Impact of independent variables on the ratio of homozygous to heterozygous variant calls.** Slope coefficients resulting from multiple variable regression analysis are shown, considering as dependent variable the ratios of observed homozygous:heterozygous to true homozygous:heterozygous variant call ratios, while GC content, read length, damage level and coverage depth were treated as independent variables. Values of 1, 2 and 3 were assigned to no-, low- and high-damage categories, respectively. Values not significant at Pr < 0.05 unless otherwise indicated; ** signifies 0.001 < Pr < =0.01; *** signifies Pr < =0.001. Additional file 5:
**Schematic of generation of simulated reference/sample sequence pairs and replicate sample read pools.** A) For each of three GC% levels, three replicate reference/sample pairs were generated using INDELible v1.03 at low divergence and three replicate pairs were generated at high divergence, for a total of 18 reference/sample pairs; each sequence was ca. 10001000 bp in length. B) For each replicate “sample” sequence, simulated Illumina 100 bp single-end read pools were generated using ART v1.5.0 (art_Illumina Q version) to 600x coverage depth; read pools were then trimmed to size distributions with average lengths 40, 60 and 80 bp (C). D) Fragmentation bias and 5′ C-T and 3′ G-A misincorporations were added at high or low frequencies for “damage” read pools. E) Final read pools were created by randomly drawing reads to different coverage depths; these read pools were mapped back to each “sample’s” original “reference”, and called variants were compared to actual variants. Additional file 6:
**Frequency of selection for fragmentation biases.** Calculations for the frequency of selection of reads based on the nucleotide identity immediately prior to (5′) or after (3′ to) read starts and ends, respectively. Additional file 7:
**Example command lines used for mapping sample read pools to corresponding reference sequences.**
