# Supplementary figures and images for: Impacts of low coverage depths and post-mortem DNA damage on variant calling: a simulation study
Source: BMC Genomics. 2015 Jan 23;16(1):19. doi: 10.1186/s12864-015-1219-8 (PMC4312461; doi:10.1186/s12864-015-1219-8)

Additional File 1

A)

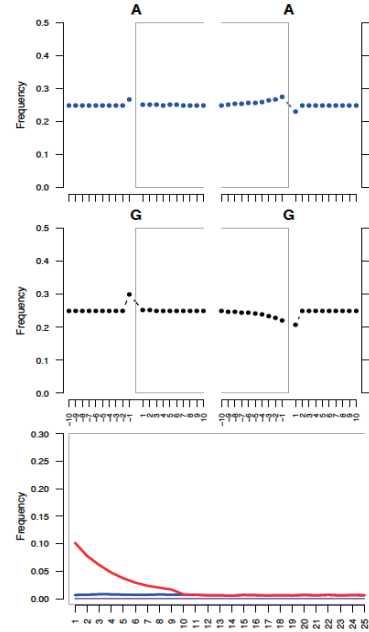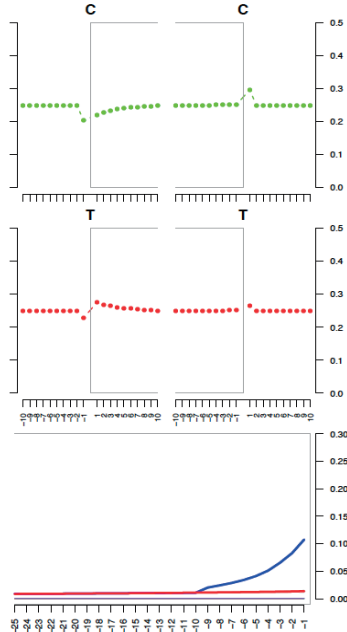

B)

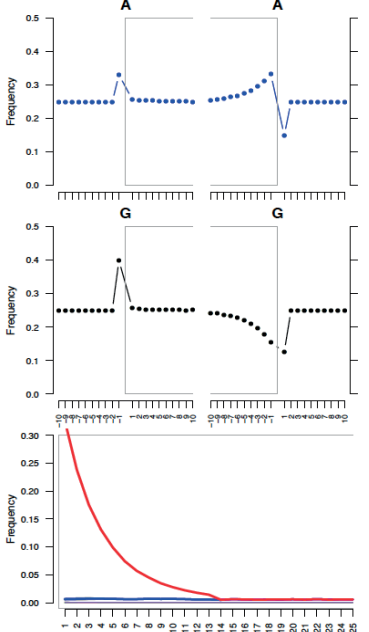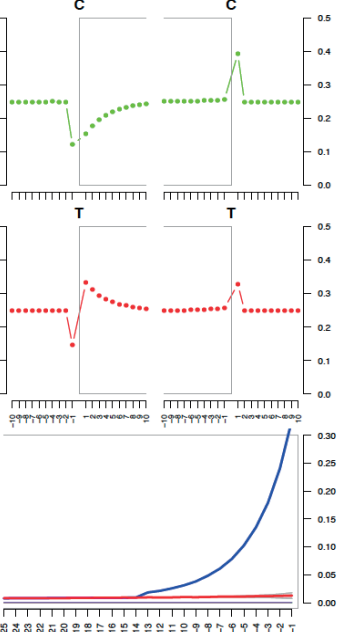

C)

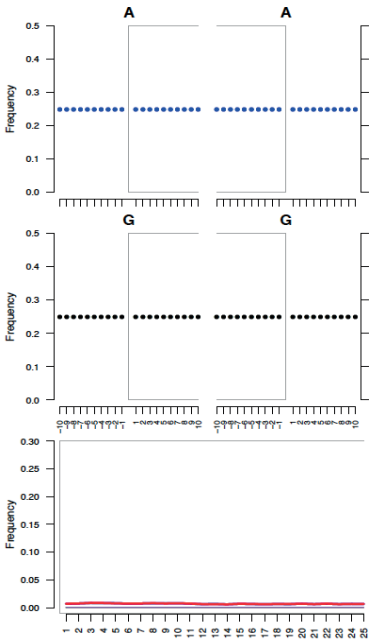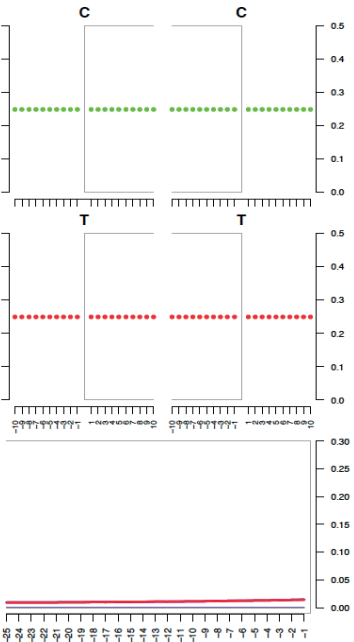

Supplement: Additional file 1: — Examples of typical fragmentation bias and misincorporation patterns. Example fragmentation and misincorporation patterns for A) low damage sample read pool, B) high damage read pool and C) undamaged read pool. Charts shown are for sample read pool with 50% GC content and 60 bp average read length. Analyses performed using mapDamage v2.0. [file 12864_2015_1219_MOESM1_ESM.pdf]
