# Supplementary material for: Impacts of low coverage depths and post-mortem DNA damage on variant calling: a simulation study
Source: BMC Genomics. 2015 Jan 23;16(1):19. doi: 10.1186/s12864-015-1219-8 (PMC4312461; doi:10.1186/s12864-015-1219-8)
Supplement: Additional file 2: — Summaries of misincorporation frequencies of mapped reads. A) Ratios of observed versus expected misincorporation rates at 5′ and 3′ sequence ends. Expected values were based on frequency of transition damages incorporated during read synthesis (see Methods). ** signifies confidence interval of value does not include 1 at 0.001 < Pr < 0.01; *** signifies confidence interval of value does not include 1 at Pr < 0.001; remaining values not significant at Pr < 0.05. B) Slope coefficients from multiple variable regression are shown, treating as dependent variables the average misincorporation frequencies for mapped reads at all 5′ and 3′ sequence read positions with elevated misincorporation frequencies (all elevated) or solely the 5′- or 3′-most positions of reads; GC content, read length, damage level and coverage depth are independent variables. Values of 1, 2 and 3 were assigned to no-, low- and high-damage read pools, respectively. Values not significant at Pr < 0.05 unless otherwise indicated; * signifies 0.01 < Pr < =0.05; *** signifies Pr < =0.001. Results in B) are shown solely for best-fit linear models except for “damage” for 5′ and 3′ “all elevated positions” at both low and high divergence. For these cases, a slight improvement in correlation was seen using an exponential model, and slope coefficients for both models are shown as: exponential coefficient/linear coefficient. [file 12864_2015_1219_MOESM2_ESM.pdf]

## Additional File 2

## A)

| Divergence | Damage | C-T observed / expected, all 5' elevated | C-T observed / expected, 5'-most position | G-A observed / expected, all 3' elevated | G-A observed / expected, 3'-most position | 5' C-T / 3'G-A, all elevated positions | 5' C-T / 3'G-A, 5'- and 3'-most positions |
|---|---|---|---|---|---|---|---|
| low | low | 1.065*** | 0.877*** | 1.298*** | 0.948*** | 0.844*** | 0.922*** |
| | high | 0.920*** | 0.850*** | 1.049** | 0.855*** | 0.899*** | 0.993** |
| high | low | 1.038 | 0.791*** | 1.239*** | 0.868*** | 0.853*** | 0.907*** |
| | high | 0.875*** | 0.764*** | 0.990 | 0.783*** | 0.903*** | 0.976*** |

**B)**

| Divergence | Read end | Position | %GC | read length | damage | coverage depth |
|---|---|---|---|---|---|---|
| low | 5’ | all elevated | $-1.02 \times 10^{-4}$ | $-3.31 \times 10^{-4}$ *** | $0.003e^{1.036(damage)}$*** / $5.93 \times 10^{-2}$ *** | $-4.74 \times 10^{-6}$ |
| | | 5’-most | $-3.06 \times 10^{-4}$*** | $-1.10 \times 10^{-3}$ *** | $2.19 \times 10^{-1}$ *** | $-3.60 \times 10^{-5}$ |
| | 3’ | all elevated | $-1.09 \times 10^{-4}$ | $-3.76 \times 10^{-5}$ | $0.007e^{0.769(damage)}$*** / $5.81 \times 10^{-2}$ *** | $-1.08 \times 10^{-6}$ |
| | | 3’-most | $-3.06 \times 10^{-4}$*** | $-8.76 \times 10^{-4}$ *** | $2.13 \times 10^{-1}$ *** | $2.88 \times 10^{-7}$ |
| high | 5’ | all elevated | $-1.52 \times 10^{-4}$* | $-3.40 \times 10^{-4}$ *** | $0.005e^{0.864(damage)}$*** / $5.21 \times 10^{-2}$ *** | $-8.76 \times 10^{-6}$ |
| | | 5’-most | $-3.03 \times 10^{-4}$*** | $-1.11 \times 10^{-3}$*** | $1.97 \times 10^{-1}$ *** | $-8.57 \times 10^{-6}$ |
| | 3’ | all elevated | $-1.61 \times 10^{-4}$* | $-8.61 \times 10^{-5}$ | $0.009e^{0.678(damage)}$*** / $5.16 \times 10^{-2}$ *** | $-1.22 \times 10^{-5}$ |
| | | 3’-most | $-3.63 \times 10^{-4}$*** | $-9.04 \times 10^{-4}$ *** | $1.95 \times 10^{-1}$ *** | $-4.33 \times 10^{-5}$ |
