# Supplementary material for: Impacts of low coverage depths and post-mortem DNA damage on variant calling: a simulation study
Source: BMC Genomics. 2015 Jan 23;16(1):19. doi: 10.1186/s12864-015-1219-8 (PMC4312461; doi:10.1186/s12864-015-1219-8)
Supplement: Additional file 3: — Regression of read length on percent of reads mapped at different damage and divergence levels. Slopes of linear regressions and R2 values are shown, considering as the dependent variable the percent of reads mapped from a pool, and read length treated as independent variable. [file 12864_2015_1219_MOESM3_ESM.pdf]

Additional File 3

divergence level	damage level	slope of linear regression	R^2 value of linear regression
low	no damage	-1.0265	0.88658
	low damage	-1.0636	0.89829
	high damage	-1.0785	0.91939
high	no damage	-1.2930	0.97598
	low damage	-1.2635	0.98029
	high damage	-1.1383	0.98760
