# Supplementary material for: Impacts of low coverage depths and post-mortem DNA damage on variant calling: a simulation study
Source: BMC Genomics. 2015 Jan 23;16(1):19. doi: 10.1186/s12864-015-1219-8 (PMC4312461; doi:10.1186/s12864-015-1219-8)
Supplement: Additional file 4: — Impact of independent variables on the ratio of homozygous to heterozygous variant calls. Slope coefficients resulting from multiple variable regression analysis are shown, considering as dependent variable the ratios of observed homozygous:heterozygous to true homozygous:heterozygous variant call ratios, while GC content, read length, damage level and coverage depth were treated as independent variables. Values of 1, 2 and 3 were assigned to no-, low- and high-damage categories, respectively. Values not significant at Pr < 0.05 unless otherwise indicated; ** signifies 0.001 < Pr < =0.01; *** signifies Pr < =0.001. [file 12864_2015_1219_MOESM4_ESM.pdf]

Additional File 4

Divergence		%GC	read length	damage	coverage depth
low	indels	-0.0047	0.0088	0.0012	-0.0814***
	SNPs	0.0131	-0.0076	0.1128	-0.2443***
high	indels	-0.0023	-0.0007	-0.0458	-0.0653***
	SNPs	0.0048	-0.0137***	0.0524	-0.1837***
