# Supplementary material for: Impacts of low coverage depths and post-mortem DNA damage on variant calling: a simulation study
Source: BMC Genomics. 2015 Jan 23;16(1):19. doi: 10.1186/s12864-015-1219-8 (PMC4312461; doi:10.1186/s12864-015-1219-8)

Additional File 5

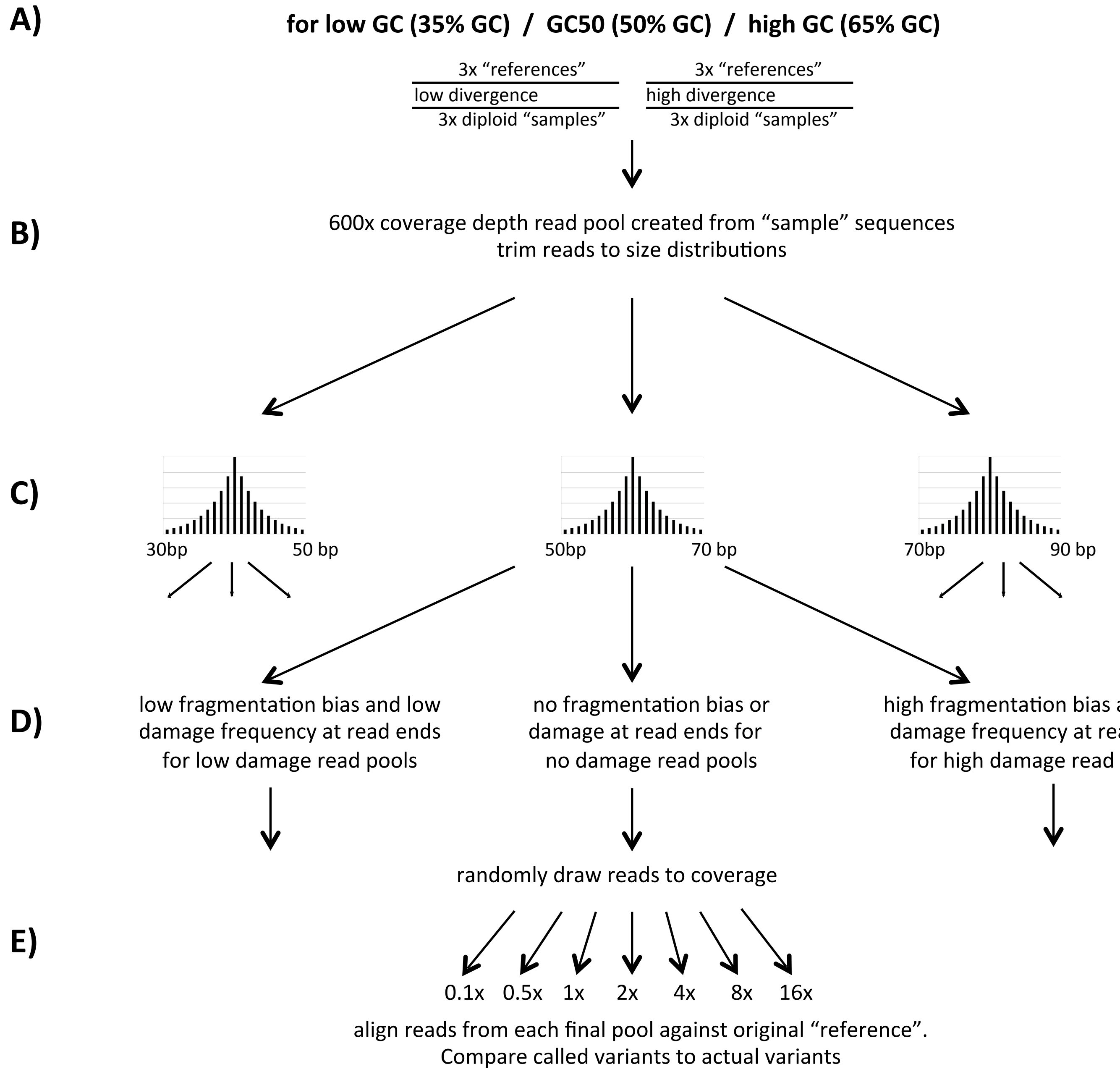

Supplement: Additional file 5: — Schematic of generation of simulated reference/sample sequence pairs and replicate sample read pools. A) For each of three GC% levels, three replicate reference/sample pairs were generated using INDELible v1.03 at low divergence and three replicate pairs were generated at high divergence, for a total of 18 reference/sample pairs; each sequence was ca. 10001000 bp in length. B) For each replicate “sample” sequence, simulated Illumina 100 bp single-end read pools were generated using ART v1.5.0 (art_Illumina Q version) to 600x coverage depth; read pools were then trimmed to size distributions with average lengths 40, 60 and 80 bp (C). D) Fragmentation bias and 5′ C-T and 3′ G-A misincorporations were added at high or low frequencies for “damage” read pools. E) Final read pools were created by randomly drawing reads to different coverage depths; these read pools were mapped back to each “sample’s” original “reference”, and called variants were compared to actual variants. [file 12864_2015_1219_MOESM5_ESM.pdf]
