# Supplementary material for: Impacts of low coverage depths and post-mortem DNA damage on variant calling: a simulation study
Source: BMC Genomics. 2015 Jan 23;16(1):19. doi: 10.1186/s12864-015-1219-8 (PMC4312461; doi:10.1186/s12864-015-1219-8)
Supplement: Additional file 7: — Example command lines used for mapping sample read pools to corresponding reference sequences. [file 12864_2015_1219_MOESM7_ESM.pdf]

Additional File 7

```
bwa aln -l 1024 -f ./sample.1x.sai ./reference.fasta ./sample.1x.fq

bwa samse -r  @RG"\t"ID:simulated"\t"SM:sample.1x -f./sample.1x.sam ./reference.fasta ./sample.1x.sai ./sample.1x.fq

samtools view -bS ./sample.1x.sam > ./sample.1x.bam

samtools sort ./sample.1x.bam ./sample.1x.bam_sorted

samtools index ./sample.1x.bam_sorted.bam

java -Xmx4g -jar ./GenomeAnalysisTK.jar -T PrintReads -R ./reference.fasta -I ./sample.1x.bam_sorted.bam -o ./sample.1x.sorted.qualrecal.bam

java  -Xmx4g -jar ./GenomeAnalysisTK.jar -T RealignerTargetCreator -R ./reference.fasta -I ./sample.1x.bam_sorted.bam -o ./sample.1x.sorted.indel.intervals

java -Xmx4g -jar ./GenomeAnalysisTK.jar -T IndelRealigner -R ./reference.fasta -I ./sample.1x.sorted.qualrecal.bam -targetIntervals ./sample.1x.sorted.indel.intervals \
-o ./sample.1x.sorted.qualrecal.indelrealign.bam

samtools flagstat ./sample.1x.sorted.qualrecal.indelrealign.bam > ./sample.1x.sorted.qualrecal.indelrealign.bam.flagstat.txt

samtools index ./sample.1x.sorted.qualrecal.indelrealign.bam

samtools mpileup -Bg -f ./reference.fasta ./sample.1x.sorted.qualrecal.indelrealign.bam > ./sample.1x.sorted.qualrecal.indelrealign.bam.pileup

bcftools view -LNcgv ./sample.1x.sorted.qualrecal.indelrealign.bam.pileup > ./sample.1x.sorted.qualrecal.indelrealign.raw.bcf

vcfutils.pl varFilter -Q 20 -D 30 -d 1 ./sample.1x.sorted.qualrecal.indelrealign.raw.bcf > ./sample.1x.sorted.qualrecal.indelrealign.q20.vcf
```
